# Molecular Epidemiology of *Coxiella burnetii* in Boer Goats and Their Farm Environment in South Korea with a One Health Approach

**DOI:** 10.3390/ani15101498

**Published:** 2025-05-21

**Authors:** You-Jeong Lee, Beoul Kim, Yong-Myung Kang, Dongmi Kwak, Min-Goo Seo

**Affiliations:** College of Veterinary Medicine & Institute for Veterinary Biomedical Science, Kyungpook National University, 80 Daehak-ro, Buk-gu, Daegu 41566, Republic of Korea; wowgirlsgood@naver.com (Y.-J.L.); kbjjhnm@naver.com (B.K.); kamaboy88@knu.ac.kr (Y.-M.K.); dmkwak@knu.ac.kr (D.K.)

**Keywords:** boer goat, *Coxiella burnetii*, clinical case, environmental contamination, one health

## Abstract

Q fever, caused by *Coxiella burnetii*, is a zoonotic disease that poses significant risks to both livestock productivity and human health. This is the first molecular and epidemiological study of *C. burnetii* infections on a Boer goat farm in South Korea, encompassing clinical cases in animals and humans, as well as environmental contamination. The investigation was initiated following abortion cases at a farm that co-raised Boer goats and cattle. Samples—including vaginal swabs, aborted fetuses, blood, tissues, feces, dust, and soil—were tested using PCR, confirming *C. burnetii* infection from multiple sources. Genotyping through Multiple Locus Variable Number Tandem Repeat Analysis and Multispacer Sequence Typing revealed molecular epidemiological links between farm-detected strains and those previously identified in human cases from South Korea, France, and the UK, suggesting possible zoonotic transmission. *C. burnetii* was also found in dust, soil, feces, and in farmworkers, indicating ongoing environmental and occupational exposure. These findings emphasize the role of environmental contamination in disease persistence and the need for improved biosecurity and coordinated surveillance. This study supports a One Health approach and offers meaningful implications for public health policy, particularly in establishing effective strategies for monitoring and controlling Q fever in livestock farms and surrounding communities.

## Simple Summary

Q fever is a zoonotic disease that can spread from animals to humans and is caused by *Coxiella burnetii*. It can cause abortions in goats and flu-like symptoms in humans. In this study, we investigated an outbreak of Q fever on a Boer goat farm in South Korea. After several goats experienced abortions, we tested animals and farmworkers for infection and environmental samples for contamination. We found evidence of *C. burnetii* infection in many female Boer goats and environmental contamination in farm dust, soil, and feces. Even some farmworkers became sick. By analyzing the genetic information of the bacteria, we discovered that it matched strains previously found in humans from South Korea, France, and the UK. This suggests that the same strains may be shared between animals and humans. Our study highlights how Q fever can persist in the farm environment and continue spreading if farms do not follow proper cleaning and infection control measures. We stress the importance of a “One Health” approach, where animal, human, and environmental health are managed together. This can help reduce disease transmission, protect livestock, and keep people safe.

## 1. Introduction

Q fever is a zoonotic disease caused by the intracellular bacterium *Coxiella burnetii*, which poses a significant public health risk owing to its ability to infect animals and humans [1]. Q fever primarily affects livestock, including cattle, sheep, and goats, but can also spread through wildlife, pets, birds, and ticks [2]. Although the infection often presents subclinically or with no apparent signs, studies have shown that it can impair reproductive performance, including reduced fertility, abortion, and complications during parturition, ultimately resulting in reproductive losses such as abortion and stillbirth in livestock [3]. Humans typically contract the infection by inhaling aerosolized bacteria from contaminated birth products or bodily fluids of infected animals [4]. Q fever often shows no clinical signs in small ruminants such as goats and sheep, but it can still lead to serious reproductive outcomes, particularly abortion and stillbirth in late pregnancy [5]. In goat herds, Q fever-related abortion rates can reach up to 90%, posing a major threat to livestock farming [6]. *C. burnetii* spreads primarily through bacterial shedding from infected animals, particularly through the placenta and fetal fluids during abortion or normal delivery. These materials contain high bacterial loads, heightening the risk of environmental contamination and disease transmission [7]. Aerosolized *C. burnetii* can disperse over long distances, infecting individuals far from the initial infection site [8].

Over the past decade in South Korea, *C. burnetii* infections have been reported in various livestock, including cattle, goats (both native Korean goats and Boer goats), pigs, and horses [9,10,11,12,13]. However, most of these studies relied on serological assays, such as ELISA or conventional PCR techniques, without further molecular characterization of the pathogen. Additionally, both *Coxiella*-like endosymbionts [14] and *C. burnetii* [15] have been identified in ticks. According to global studies, the Netherlands experienced the largest recorded Q fever outbreak in humans, beginning in 2007, with abortions on dairy goat and sheep farms between 2005 and 2009, identifying these small ruminants as the primary source [16]. Recent studies have also detected *C. burnetii* infections in sheep and goats in Greece [17] and in goats in Germany [18], highlighting the widespread distribution of the disease.

In South Korea, the consumption of dog meat was recently banned. As a result, the demand for alternative protein sources, particularly goat meat, has increased markedly. Especially, Boer goats have gained attention as a preferred breed for meat production due to their rapid growth, meat quality, and adaptability. This shift has led to the expansion of Boer goat farming across the country. Against this backdrop, the present study aims to investigate the presence of *C. burnetii* in a Boer goat farm in South Korea, focusing on clinical cases and environmental contamination. Specifically, it assesses infection rates among Boer goats, examines contamination levels in the farm environment, and underscores the need for biosecurity practices. Understanding the potential role of *C. burnetii* in reproductive failures and its persistence in the environment highlights the importance of adopting a One Health approach—integrating animal, environmental, and human health strategies—for effective prevention and control of Q fever in livestock farming systems. Unlike previous domestic studies that focused primarily on diagnostic detection, this study advances the field by incorporating genotypic analysis—including Multiple Locus Variable Number Tandem Repeat Analysis (MLVA) and Multispacer Sequence Typing (MST)—along with environmental surveillance, clinical case monitoring, and follow-up assessments after treatment. This integrated approach provides a comprehensive understanding of Q fever transmission dynamics in South Korean goat farms.

## 2. Materials and Methods

### 2.1. Ethical Approval

This study followed ethical guidelines and was approved by the Institutional Animal Care and Use Committee of Kyungpook National University (approval No. KNU 2024-0281). Noninvasive samples (feces, dust, and soil) were collected, while licensed veterinarians obtained vaginal swabs and blood samples. All procedures adhered to national and institutional animal welfare regulations. No human participants were enrolled, and no clinical procedures or data collection involving humans were conducted by the research team. Therefore, Institutional Review Board approval for human subjects was not required.

### 2.2. Case History and Sample Collection

**Figure 1:**
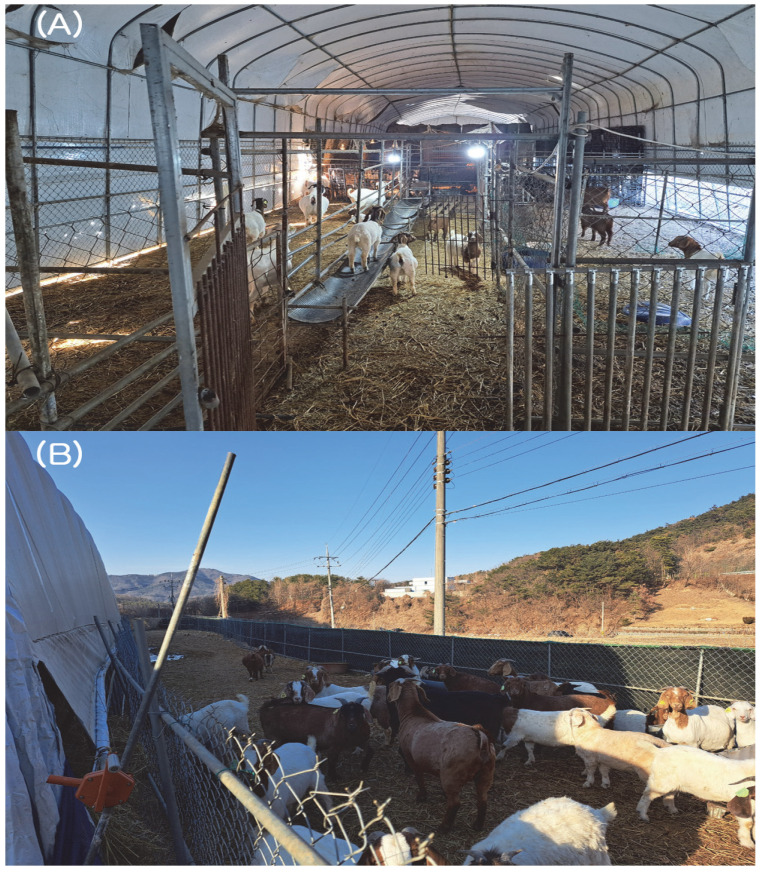
The Boer goat farm was infected with *Coxiella burnetii*. (**A**) Interior and (**B**) exterior views of the Boer goat farm stalls. The barns were converted from vinyl greenhouse structures originally intended for crop production. Goats were housed without physical barriers on soil floors covered with rice straw. The facility lacked key biosecurity features, such as disinfection zones and controlled access, and had no mechanical ventilation system.

In early November 2024, a farm located in Gyeongbuk Province, South Korea, housed a total of 155 animals, including 133 mixed Boer goats (55 in the lower barn and 78 in the upper barn) and 22 cattle. The animals were kept under substandard housing conditions (Figure 1). The barns were constructed using vinyl greenhouse structures originally intended for crop cultivation rather than livestock housing. Goats were housed without physical barriers, and the soil-based floors were covered with rice straw litter. The facility lacked basic biosecurity infrastructure, including disinfection zones, controlled entry points, and isolation areas. Furthermore, no mechanical ventilation systems were present, resulting in poor air circulation. At that time, all animals were vaccinated against foot-and-mouth disease and lumpy skin disease using government-approved commercial vaccines. Following the vaccination, one adult female Boer goat died, and another had a miscarriage. Tissue samples from the deceased Boer goat (uterus, liver, lungs, spleen, kidneys) and from the aborted fetus (placenta, liver, lungs, spleen, kidneys, muscle) were collected during necropsy and submitted to the laboratory at the College of Veterinary Medicine, Kyungpook National University, for differential diagnosis (DDx), including potential vaccine-related effects. Although these events occurred shortly after vaccination, the possibility of vaccine interference was investigated and ruled out. Due to confidentiality considerations and to avoid misinterpretation, specific product names are not disclosed in this manuscript. Postmortem examination of the deceased Boer goat revealed pneumonia-related lung lesions, uterine rupture, and peritonitis. Among two initial samples—one from the deceased goat and one from the first aborted fetus—differential PCR tests for ten major reproductive pathogens showed no evidence of vaccine-related pathology. *C. burnetii* was the only pathogen identified. Two days later, another adult female Boer goat suffered a miscarriage. Tissue samples from the second aborted fetus (placenta, liver, lungs, spleen, kidneys, muscle) were similarly collected and submitted for laboratory analysis. Ten differential diagnostic tests for abortion-related pathogens were performed on this additional case as well. PCR results were negative for all tested pathogens except *C. burnetii*. Overall, *C. burnetii* DNA was detected in the uterus and liver tissues of the deceased goat and in all tested tissues (placenta, liver, lungs, spleen, kidneys, muscle) from both aborted fetuses.

In late November, the first-round expanded screening test was performed on the farm. Vaginal swab samples were collected from 119 female Boer goats and 22 female cattle, while blood samples were collected from 14 male Boer goats. As cattle were co-housed with goats on the farm, they were also included in the whole-herd screening to evaluate the possibility of interspecies transmission of *C. burnetii.* All samples were analyzed using PCR to detect *C. burnetii*. Due to the urgent need to screen the entire herd and limitations in field conditions, environmental sampling was not feasible during this initial investigation. For further molecular characterization, PCR-positive samples were analyzed using MLVA and MST. After diagnosis, treatment with oxytetracycline hydrochloride was initiated. This antibiotic is effective against bacteria sensitive to oxytetracycline and is sometimes used for Q fever, though tetracycline or doxycycline are more commonly recommended. For Boer goats, the dosage was set at 14.8 g per goat, based on an average weight of 40 kg, and administered through drinking water. The treatment plan for Q fever-positive animals was scheduled for 14 days.

In late January 2025, a second-round screening test was conducted on the farm. Since only female Boer goats tested positive in the first round, the second round focused exclusively on females. Although 119 female Boer goats were initially targeted, four had died since the first round, and vaginal swab samples were ultimately collected from 115 female Boer goats. In addition, one aborted fetus was discovered in the lower barn during the second round. Tissue samples from this fetus (placenta, liver, lungs, spleen, kidneys, muscle) were collected. All samples were analyzed using PCR to detect *C. burnetii*. Furthermore, environmental samples were collected separately from both the upper and lower barns to investigate potential contamination sources. In each barn, dust samples were collected using swabs from multiple locations, including the entrance, fence, maternity pen, feed storage, composting area, barn interior, feed trough, and floor. Soil samples were obtained from various ground-level sites (entrance, fence, maternity pen, barn, and floor), and fecal samples were gathered from multiple spots on the barn floor. For each barn, dust, soil, and fecal samples were pooled by type into individual 50 mL tubes, resulting in three pooled environmental samples per barn. In total, six pooled environmental samples (three from the upper barn and three from the lower barn) were obtained and individually tested for *C. burnetii* using PCR.

### 2.3. DNA Extraction

DNA was extracted from various sample types using specific extraction kits. Animal samples, including tissue, blood, and vaginal swabs, were extracted using a DNeasy Blood & Tissue Kit (Qiagen, Melbourne, Australia). For environmental samples, different extraction methods were employed. DNA from dust samples was extracted employing the Clear-STM Quick DNA Extraction Kit (InVIRUStech, Gwangju, South Korea), while DNA from feces and soil samples was extracted using the Beniprep Soil/Fecal DNA Extraction Kit (InVIRUStech).

### 2.4. Abortion-Related Diseases Screening Method

To identify the cause of the abortions on the farm, ten differential diagnostic tests were performed on three samples: one from a deceased Boer goat and two from aborted fetuses. Both real-time PCR and conventional PCR methods were used to detect pathogens associated with abortion. For real-time PCR screening, commercial PowerChek™ Real-time PCR kits (Kogenebiotech, Seoul, South Korea) were used to detect *Toxoplasma gondii*, *Listeria monocytogenes*, *Campylobacter fetus*, *Salmonella dublin*, and *Brucella abortus*.

For conventional PCR screening, commercial diagnostic kits were used to detect Akabane virus, Chuzan virus, *Neospora caninum*, and *Leptospira* spp. The following kits were used: VDx^®^ Bovine Akabane/Aino MP RT-PCR Kit (MedianDiagnostics, Chuncheon, South Korea), VDx^®^ Bovine Chuzan/BEF/Ibaraki MP RT-PCR Kit (MedianDiagnostics), LiliF™ Neospora PCR Kit (IntronBiotechnology, Seongnam, South Korea), and LiliF™ LEPTO PCR Kit (IntronBiotechnology). For *C. burnetii*, conventional PCR was performed.

### 2.5. Conventional PCR Protocol for C. burnetii Detection

Conventional PCR targeting the IS1111 transposase gene of *C. burnetii* was conducted using the AccuPower PCR Premix kit (Bioneer, Daejeon, South Korea) with Trans 1 and Trans 2 primers [19]. The expected amplicon size was 687 bp. Specifically, a sample of *C. burnetii* in pigs [10] from our previous studies in South Korea was used as a positive control. A negative control, which did not contain a DNA template, was also included to verify the absence of contamination.

### 2.6. MLVA Genotyping Procedure

MLVA genotyping was performed on PCR-positive samples using the AccuPower Hotstart PCR Premix kit (Bioneer). Six variable number tandem repeat markers—Ms23, Ms24, Ms27, Ms28, Ms33, and Ms34—were analyzed using specific primers and following the protocol [20].

### 2.7. MST Genotyping Procedure

MST genotyping was performed using the AccuPower PCR Hotstart Premix kit (Bioneer). Ten spacers—Cox2, Cox5, Cox18, Cox20, Cox22, Cox37, Cox51, Cox56, Cox57, and Cox61—were analyzed using specific primers and following the protocol [20].

### 2.8. DNA Sequencing and Phylogenetic Analysis

Positive samples were sequenced at Macrogen (Seoul, South Korea) using designated primer sets. Sequence alignments were performed with CLUSTAL Omega (v1.2.1), and sequence editing was conducted using BioEdit (v7.2.5). Phylogenetic trees were constructed using MEGA (v6.0) with the maximum likelihood method and Kimura two-parameter model. Bootstrap analysis (1000 replicates) was performed to assess tree stability.

### 2.9. Statistical Analyses

Statistical analyses were performed using GraphPad Prism version 5.04 (GraphPad Software Inc., La Jolla, CA, USA). The Fisher’s exact test was used for 2 × 2 tables, and the chi-square value (χ^2^) and degrees of freedom (df) were reported. *p* < 0.05 was considered statistically significant, and 95% confidence intervals (CI) were calculated for all estimates.

## 3. Results

### 3.1. Results of Abortion-Related Disease Screening

Ten differential diagnostic tests were conducted on three samples: one from a deceased Boer goat and two from aborted fetuses. Of the tested pathogens, only *C. burnetii* was detected, confirming Q fever infection in all three cases.

### 3.2. C. burnetii Detection by Conventional PCR

In the first-round expanded screening, 155 samples were analyzed, including 22 cattle and 133 Boer goats. Of these, 31 samples (20.0%) tested positive for *C. burnetii* (Table 1). All positive samples came from female Boer goats, with an infection rate of 26.1% (31/119). None of the 22 cattle or 14 male Boer goats tested positive. Among the female Boer goats, all 78 individuals housed in the upper barn tested negative, while 31 out of 41 individuals (75.6%, CI: 62.5–88.8) in the lower barn tested positive. The infection rate in the lower barn was significantly higher than that in the upper barn (χ^2^ = 77.389, df = 1, *p* < 0.0001).

In the second-round screening, 122 samples were analyzed, including 115 female Boer goats, one aborted fetus, and six environmental samples (soil, feces, and dust). Among these, a total of 43 samples (35.2%) tested positive (Table 2). Of the positive samples, 37 were from female Boer goats, yielding a positivity rate of 32.2% (37/115). The lower barn showed a significantly higher infection rate (36/38, 94.7%, 95% CI: 87.6–100) than the upper barn (1/77, 1.3%, 95% CI: 0–3.9), with a statistically significant difference (χ^2^ = 101.794, df = 1, *p* < 0.0001). Environmental contamination was also observed, with *C. burnetii* detected in five samples: one from the soil in the upper barn, two from feces collected from both barns, and two from dust samples from both barns. Additionally, *C. burnetii* was also identified in the aborted fetus from the lower barn.

### 3.3. MLVA Genotyping Results

MLVA genotyping was performed on all PCR-positive samples from both screening rounds. Among the 74 PCR-positive samples, a single MLVA genotype was identified, showing a consistent tandem-repeat pattern of 4-18-3-5-8-2 across all six loci (MS23, MS24, MS27, MS28, MS33, and MS34). Since no variation was observed among samples, this consensus pattern was presented as the representative MLVA genotype. Comparison with the MLVA genotype database (https://microbesgenotyping.i2bc.paris-saclay.fr/databases, accessed on 10 February 2025) showed that this pattern closely matched one previously found in a human heart sample from France in 1993. The database version used for comparison was Coxiella burnetii_v3_1, accessed at the end of December 2024 and again in early February 2025.

### 3.4. MST Genotyping Results

**Figure 2:**
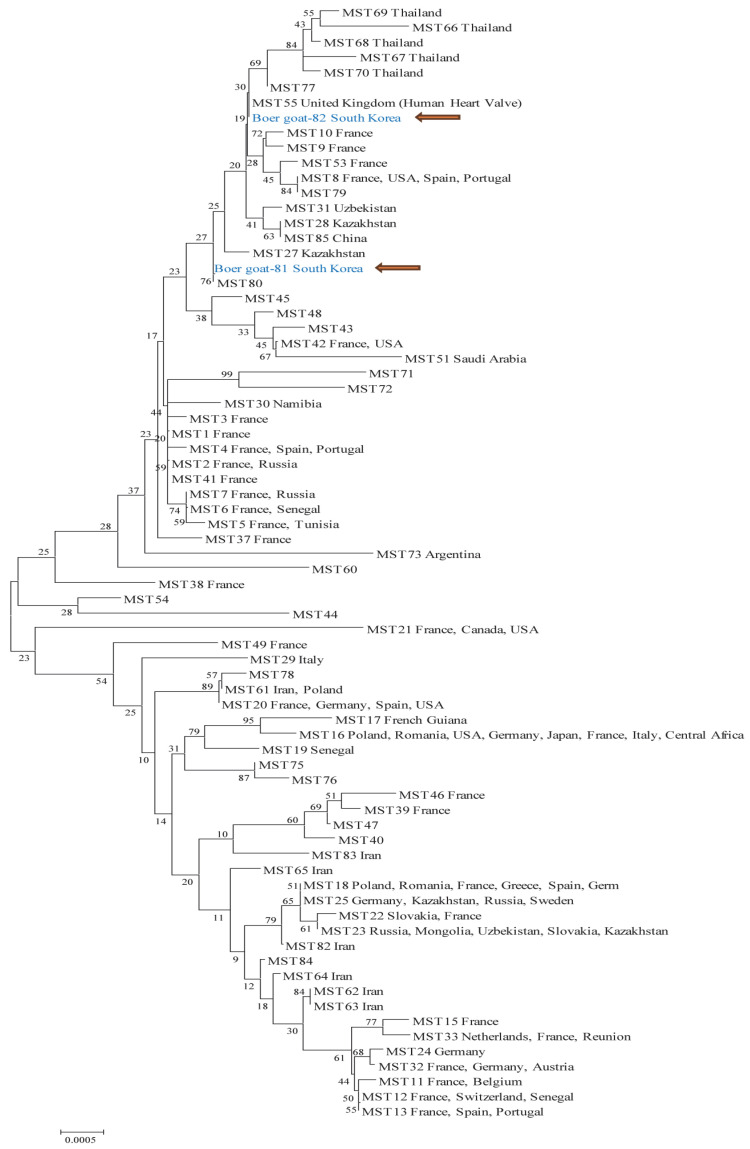
A phylogenetic tree illustrating the relationships between different MST groups of *Coxiella burnetii*. All PCR-positive samples were sequenced, and sequences with identical MST profiles were grouped and represented by a single sequence. Two distinct MST genotypes (MST55 and MST80) were identified from Boer goats in South Korea. The tree was constructed using the maximum likelihood method based on sequences obtained from vaginal swab samples and reference MST groups. The analysis was performed using the Kimura 2-parameter model with a γ distribution and 1000 bootstrap replicates in MEGA6 software. The sequences analyzed in this study are indicated by orange arrows.

MST genotyping was conducted on all PCR-positive samples, analyzing 10 genetic markers (Cox2, Cox5, Cox18, Cox20, Cox22, Cox37, Cox51, Cox56, Cox57, and Cox61), and sequences with identical MST patterns were grouped. Two distinct representative genotypes were identified and presented in Figure 2 to reflect the observed diversity. Boer goat 82 exhibited a spacer-type pattern of 5-4-9-5-8-5-2-3-4-6, closely matching MST55, a genotype previously found in a human heart sample from the UK. Boer goat 81 displayed a spacer-type pattern of 5-4-8-5-8-5-2-3-4-6, aligning with MST80, a genotype with no additional information available. MST genotypes were determined using the MST database and online BLAST tool (v. 2.14.1) available at https://ifr48.timone.univ-mrs.fr/mst/coxiella_burnetii/blast.html, accessed on 10 February 2025. Additionally, the resulting MST sequences were compared with publicly available reference sequences in the NCBI GenBank database using the BLAST tool. This analysis revealed a high similarity to human-derived *C. burnetii* sequences (accession numbers CP107268 and CP107247), which were isolated from blood samples collected in Cheongju and Iksan, South of Korea, in 2016.

### 3.5. Molecular and Phylogenetic Analyses

**Figure 3:**
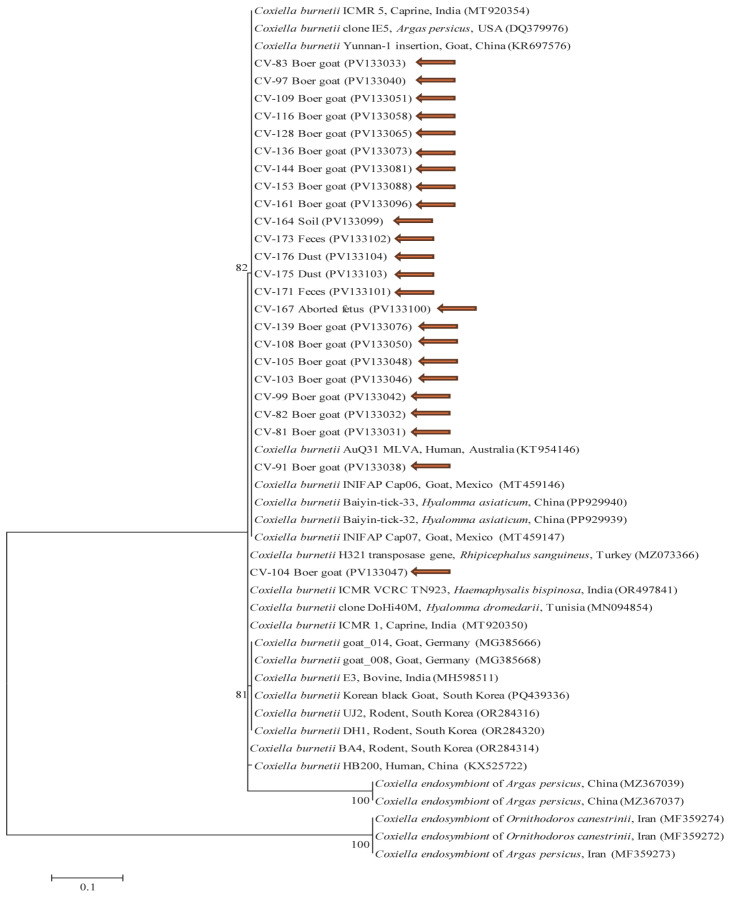
A phylogenetic tree was constructed based on the IS1111 sequences of *Coxiella burnetii* using the maximum likelihood method. Among the 74 PCR-positive samples, 24 representative sequences were selected. Identical sequences were grouped and represented by a single sequence, while all unique sequences were included individually to reflect genetic diversity. The orange arrows highlight the sequences analyzed in this study. GenBank accession numbers for the other sequences are provided alongside their corresponding sequence names. *Anaplasma phagocytophilum* was used as the outgroup. Bootstrap support values ≥ 70% (1000 replicates) are shown on the corresponding nodes, and the scale bar represents phylogenetic distance.

A phylogenetic tree was constructed using 24 representative sequences selected from the 74 PCR-positive samples (Figure 3). Sequences that were identical were grouped, and only one representative was included; all unique sequences were included individually to reflect genetic variation. Bootstrap values ≥ 70% (based on 1000 replicates) were displayed to indicate statistically supported nodes. The 24 *C. burnetii* IS1111 transposase gene sequences exhibited 99.7–100% similarity among themselves and 99.0–100% identity with previously reported *C. burnetii* isolates in GenBank. All sequences generated in this study have been deposited in GenBank under the following accession numbers: PV133031-PV133104 (*C. burnetii* IS1111 transposase gene).

## 4. Discussion

In South Korea, the consumption of dog meat was recently banned. In February 2024, the Ministry of Agriculture, Food, and Rural Affairs enacted a law prohibiting the breeding, slaughter, and distribution of dogs for food [21]. Following the ban, the demand for goat meat surged, driving the expansion of goat farming as it became a popular alternative protein due to shifting dietary preferences. According to livestock statistics, by December 2023, 423,430 goats were being raised across 10,263 farms in South Korea. In Gyeongbuk Province, 36,731 goats were raised across 1005 farms, making up approximately 8.7% of the national goat population and 9.8% of farms nationwide [22]. Between 2013 and 2024, *C. burnetii* infections were reported in 652 goats across 25 farms in South Korea, with an average of 178.4 goats per farm and an infection rate of 16.0% [23].

A significant outbreak occurred in 2018 at a goat farm in Cheongju, where unexplained abortions and preterm deliveries led to the detection of *C. burnetii* [24]. Of the 77 goats on the farm, six pregnant goats experienced consecutive reproductive failures, while others gave birth to weak offspring. Initially suspected to be vaccine-related, subsequent testing confirmed *C. burnetii* infection in 70.1% (54/77) of goats via PCR and 81.8% (63/77) via ELISA [24]. In our study, *C. burnetii* infection rates among female Boer goats that experienced abortions ranged from 26.1 to 32.2%, lower than the 70.1% positivity rate observed in the 2018 Cheongju case. Both studies underscore the significant role of *C. burnetii* in reproductive failures and emphasize the risk of zoonotic transmission in the absence of effective biosecurity. However, a closer comparison between the two farms reveals important differences in farm management and outbreak response. The Cheongju farm exhibited more severe gaps in biosecurity practices, including delayed isolation of symptomatic animals and poor hygiene, such as blood-stained vaginal discharge left on the barn floor. No immediate diagnostic or quarantine measures were implemented after the abortions, and human-to-human transmission occurred extensively. Six days after the abortions, the farm owner developed a high fever and systemic symptoms; his wife followed a month later. Furthermore, *C. burnetii* infection was confirmed in seven of fourteen veterinary officials who visited the site and in two laboratory technicians handling ELISA samples—even though personal protective equipment (PPE) was used [24]. In contrast, while our study farm also lacked formal biosecurity infrastructure—being housed in a vinyl greenhouse with a shared space between goats and cattle, and no mechanical ventilation or disinfection zones—basic control measures were initiated promptly. All animals were tested regardless of clinical symptoms, and infected goats were treated with antibiotics. Although PPE use among workers was insufficient, early detection and response appear to have mitigated wider transmission. Three senior farm workers who had direct contact with infected animals without wearing PPE developed flu-like symptoms, including a high fever. We advised them to seek medical attention, and they were later confirmed to be infected with *C. burnetii* by local healthcare providers. We also recommended using PPE to reduce the risk of infection. These differences in outbreak response likely contributed to the disparity in infection rates between the two farms. The comparison highlights the critical importance of timely recognition, diagnostics, and implementation of even basic biosecurity interventions in controlling disease spread. This case further reinforces the need for structured farm management training and One Health-based surveillance to prevent zoonotic spillover.

Several studies have examined the prevalence of *C. burnetii* in South Korea, particularly in goats, which are considered key reservoirs of the pathogen. Between 2016 and 2020, PCR testing revealed a prevalence of 22.7% (25/110) in Korean native goats and 6.0% (5/83) in Boer goats [9]. A previous study conducted between 2009 and 2011 found a prevalence of 19.1% (114/597) via ELISA and 9.5% (57/597) through conventional PCR in Korean native goats [11]. Studies in other regions also report varying prevalence rates in goats, which could be influenced by diagnostic methods (e.g., PCR, ELISA), as well as differences in sample sizes, study populations, and environmental conditions. Small ruminant studies of Q fever in Europe have similarly reported varying prevalence rates. In Greece, a study conducted between 2014 and 2016 analyzed samples from sheep and goats, reporting a prevalence rate of 15.7% (11/70) by real-time PCR [17]. Similarly, a 2018 study in Germany identified *C. burnetii* as the only pathogen detected on a dairy goat farm that experienced abortions, stillbirths, and weak offspring [18]. The Netherlands experienced the largest recorded Q fever outbreak in humans globally, with 3921 cases reported over four consecutive years starting in 2007. Between 2005 and 2009, Q fever triggered abortion storms on 28 dairy goat farms and 2 dairy sheep farms, with abortion rates reaching up to 80%. These small ruminants were identified as the primary source of a major Q fever outbreak in humans in The Netherlands [16]. In Asia, cases have been reported in China, with a seroprevalence of 12.2% (176/1440) in goats using ELISA from 1989 to 2013 [25]. More recently, a 2023–2024 study in Thailand found a seroprevalence of 6.4% (33/513) by ELISA, with real-time PCR analysis of vaginal swabs revealing *C. burnetii* in 17.1% (57/334) of goats [26]. These studies, although using varying diagnostic techniques (e.g., PCR vs. ELISA), all underscore the widespread presence of *C. burnetii* in goat populations across different countries, highlighting the importance of using standardized diagnostic tools for more direct comparisons. In our study, the infection rate in Boer goats (26.1–32.2%) was relatively high compared to other studies, possibly due to different diagnostic methods, sample size, or environmental factors. Differences in study design, host species, and farm management practices may also explain discrepancies in prevalence rates across regions. In this context, a comprehensive study conducted in Queensland, Australia, between 2010 and 2011 collected fecal, blood, milk, urine, and tick samples from various animals, along with soil and dust samples from multiple locations. The analysis revealed detection rates of 2.1%, 2.0%, 6.9%, 2.0%, 6.6%, 6.7%, and 6.7% in feces, soil, dust (filter), dust (vacuum swab), blood, urine, and ticks, respectively, while milk samples tested negative [27]. These findings indicate that *C. burnetii* can persist in farm environments, contributing to disease transmission. Additionally, detecting *C. burnetii* in environmental samples highlights the potential for multiple transmission routes, including airborne dispersal, indirect contact with contaminated surfaces, and tick-borne transmission. Together, these findings emphasize the need for a comprehensive disease control strategy, incorporating environmental management, farm hygiene, and biosecurity protocols to mitigate the spread of *C. burnetii* in livestock populations and prevent zoonotic transmission.

Our study examined the genetic characteristics of *C. burnetii* in South Korea using MLVA and MST typing. The MLVA analysis identified a tandem-repeat genotype (4-18-3-5-8-2) that closely matched a human heart isolate from France. This differs from the genotype (6-13-2-7-9-10) previously identified in a cattle-based study conducted in South Korea, which has also been reported in the Netherlands, France, Spain, and Poland [20]. In our study, MST analysis identified two distinct genotypes from Boer goats. Boer goat 82 showed a spacer-type pattern of 5-4-9-5-8-5-2-3-4-6, which corresponded to MST55—a spacer-type genotype within the MST classification system, previously detected in a human heart sample from the UK. Meanwhile, Boer goat 81 exhibited a unique pattern of 5-4-8-5-8-5-2-3-4-6, corresponding to MST80—a novel MST genotype not previously reported in existing databases and for which no epidemiological information is currently available. In contrast, the previously reported genotype in South Korea was MST61, which was found in cow milk from Poland and Iran and closely related to MST20, a genotype identified in France, Germany, the USA, and Spain [20]. In summary, the *C. burnetii* genotypes identified in cattle and goats show genetic similarities to strains reported in various countries, suggesting potential links between global transmission patterns. This genetic similarity to strains from France and the UK may reflect historical trade or travel connections between South Korea and these regions. Further research into the historical and epidemiological factors behind this spread will be essential for developing effective strategies for disease management and prevention. Several international research projects have explored MST genotypes of *C. burnetii*. In Iran, five MST genotypes (MST61–65) were identified in ruminants [28]. Similarly, a study in Greece (2014–2016) analyzed MLVA and MST genotypes in sheep and goats, identifying three MLVA genotypes. All of them were further typed by MST and found to belong to MST32, which had previously been detected in human cardiac valves in France and Germany, as well as in goat placenta samples from Austria [17]. These findings indicate that, while *C. burnetii* MST and MLVA genotypes often vary by geographic region and host species, some genotypes, such as MST32, have been detected across multiple regions and hosts, suggesting complex transmission dynamics between animals and humans. In our study, *C. burnetii* MST sequences from Boer goats showed genetic similarity to sequences obtained from human blood samples collected during Q fever cases in Cheongju and Iksan, South Korea, in 2016 (GenBank accession numbers CP107268 and CP107247) [29]. These comparisons were based on MST sequence alignment. This genetic similarity suggests that *C. burnetii* infections are circulating in both humans and animals in South Korea, potentially leading to persistent infections. Furthermore, these findings highlight the genetic diversity of *C. burnetii* strains in South Korea and support the possibility of zoonotic transmission.

In South Korea, government policy classifies Q fever as a type 2 livestock infectious disease. Instead of culling infected animals, management involves movement restrictions and antibiotic therapy. Surveillance is conducted through testing at 2-month intervals until the animal fully recovers. However, there is no approved vaccine for Q fever in South Korea at this time. Based on experiences from other countries, such as those in Europe and Australia, a vaccine could prove effective in future outbreaks. Antibiotic treatment is provided to infected animals, and if initial treatment fails, protocols are adjusted as recommended by veterinarians. Additional measures for airborne transmission have not been implemented, but with the growing importance of zoonotic diseases, the government may take further actions in the future. During our initial farm visit, no infections were detected in male Boer goats or cattle; however, female Boer goats showed signs of abortion. An epidemiological investigation revealed that the Boer goats were sourced from multiple regions nationwide without proper quarantine measures and housed in the lower barn, likely serving as the initial source of infection. Although both barns were managed by the same individual, the absence of biosecurity infrastructure, such as controlled movement and disinfection zones, may have facilitated indirect transmission via human activity between the barns. The initial clustering of positive cases in the lower barn underscores the need for stricter preventive protocols and highlights the importance of tracing animal movement as part of a comprehensive Q fever control strategy. In the upper barn, one female Boer goat that initially tested negative was found to be positive two months later. In the lower barn, 31 female Boer goats tested positive in the first round of testing, and in the second round, 27 of them remained positive. Of the remaining four, three died, and only one recovered and tested negative after antibiotic treatment. Although environmental samples were not collected during the first round of testing, *C. burnetii*-positive results in dust, soil, and feces during the second round highlight the role of environmental reservoirs in sustaining transmission. In Australia, *C. burnetii* was detected in environmental samples such as feces, soil, and dust [27]. Similarly, our study confirmed *C. burnetii* contamination in the farm environment, emphasizing that inadequate environmental management can promote disease persistence and further transmission. These findings highlight the critical role of effective environmental management in controlling infection spread. During the second-round visit, aborted fetuses with high concentrations of *C. burnetii* were still present on the barn floor, indicating inadequate disease control and a significant transmission risk. Furthermore, 10 goats that initially tested negative became positive in the second round of testing—one from the upper barn and nine from the lower barn. These results show that *C. burnetii* infections, initially confined to the lower barn, may have spread to the upper barn and that the pathogen remains actively circulating within the farm environment. This pattern of infection is attributed to the lack of biosecurity facilities on the farm.

Our findings suggest that without proper environmental management and strict biosecurity protocols, antibiotic treatment alone is insufficient to eliminate *C. burnetii* or to prevent reinfection and ongoing transmission. While antibiotic therapy may offer short-term benefits, *C. burnetii* is known to persist in the environment for extended periods, and reduction in bacterial burden through treatment alone may be limited. In our case, only one animal reverted to PCR-negative status two months after treatment, suggesting limited impact within that time frame. This aligns with findings by Eldin et al. (2017), who reported that antibiotics alone may not be effective for controlling persistent or chronic *C. burnetii* infections and emphasized the value of integrated management strategies that include vaccination [30]. To improve disease control, antibiotic therapy should be combined with thorough environmental decontamination and isolation of infected animals, alongside reinforced biosecurity. Our study confirmed ongoing transmission and environmental contamination even after antibiotic intervention, underscoring the importance of a One Health approach that addresses animal, human, and environmental components collectively.

## 5. Conclusions

This study provides the first comprehensive molecular and epidemiological analysis of *C. burnetii* infections in Boer goat farms in South Korea, including clinical cases in both humans and animals, as well as environmental contamination. Recurrent abortion cases in female Boer goats, ongoing fetal losses, and significant environmental contamination with *C. burnetii* were identified. The failure to implement proper cleaning and disinfection measures allowed the pathogen to persist, leading to transmission of *C. burnetii* to farmworkers. Notably, *C. burnetii* is capable of existing in a spore-like form, enabling it to survive for prolonged periods under harsh environmental conditions, which may contribute to its persistence in contaminated farm environments. Our findings indicate that *C. burnetii* is not confined to infected animals, but it can persist in various environmental sources, complicating disease control efforts. The persistent presence of *C. burnetii* in the farm environment underscores the risk of ongoing transmission. Effective prevention requires a One Health approach that integrates animal, environmental, and human health management. This strategy should incorporate not only strict biosecurity measures and continuous surveillance but also include livestock vaccination and regular health screening and immunization of farmworkers to minimize zoonotic risk. Strengthening these efforts is essential to limiting transmission, safeguarding livestock, and ensuring public health.

## Figures and Tables

**Table 1:** Positivity rates in the first-round expanded screening test were determined using conventional PCR.

Group		Location	Number of Tests	Number of Positive (%)
Boer goat	Female	Upper barn	78	0
		Lower barn	41	31 (75.6) *
	Male	Upper barn	7	0
		Lower barn	7	0
	Sub Total		133	31 (23.3)
Cattle	Female	Cattle barn	22	0
Total			155	31 (20.0)

* indicates statistically significant differences (*p* < 0.05).

**Table 2:** Positivity rates were determined via conventional PCR in the second-round screening test.

Group		Location	Number of Tests	Number of Positive (%)
Boer goat	Female	Upper barn	77	1 (1.3)
		Lower barn	38	36 (94.7) *
	Aborted fetus	Lower barn	1	1 (100)
Subtotal			116	38 (32.8)
Environmental samples	Dust	Upper barn	1	1 (100)
		Lower barn	1	1 (100)
	Soil	Upper barn	1	1 (100)
		Lower barn	1	0
	Fecal	Upper barn	1	1 (100)
		Lower barn	1	1 (100)
Total			122	43 (35.2)

* indicates statistically significant differences (*p* < 0.05).

## Data Availability

Data supporting the conclusions of this article are included within the article. The newly generated sequences were submitted to the GenBank database under the accession numbers PV133031-PV133104. The datasets used and/or analyzed during the present study are available from the corresponding author upon reasonable request.
